# Dietary Yeast Cell Wall Extract Alters the Proteome of the Skin Mucous Barrier in Atlantic Salmon (*Salmo salar*): Increased Abundance and Expression of a Calreticulin-Like Protein

**DOI:** 10.1371/journal.pone.0169075

**Published:** 2017-01-03

**Authors:** Giulia Micallef, Phillip Cash, Jorge M. O. Fernandes, Binoy Rajan, John W. Tinsley, Ralph Bickerdike, Samuel A. M. Martin, Alan S. Bowman

**Affiliations:** 1 Institute of Biological and Environmental Sciences, University of Aberdeen, Aberdeen, United Kingdom; 2 Division of Applied Medicine, Institute of Medical Sciences, University of Aberdeen, Aberdeen, United Kingdom; 3 Faculty of Biosciences and Aquaculture, Nord University, Bodø, Norway; 4 BioMar Ltd, Grangemouth Docks, Grangemouth, United Kingdom; Biomedical Research Foundation, Academy of Athens, GREECE

## Abstract

In order to improve fish health and reduce use of chemotherapeutants in aquaculture production, the immunomodulatory effect of various nutritional ingredients has been explored. In salmon, there is evidence that functional feeds can reduce the abundance of sea lice. This study aimed to determine if there were consistent changes in the skin mucus proteome that could serve as a biomarker for dietary yeast cell wall extract. The effect of dietary yeast cell wall extract on the skin mucus proteome of Atlantic salmon was examined using two-dimensional gel electrophoresis. Forty-nine spots showed a statistically significant change in their normalised volumes between the control and yeast cell wall diets. Thirteen spots were successfully identified by peptide fragment fingerprinting and LC-MS/MS and these belonged to a variety of functions and pathways. To assess the validity of the results from the proteome approach, the gene expression of a selection of these proteins was studied in skin mRNA from two different independent feeding trials using yeast cell wall extracts. A calreticulin-like protein increased in abundance at both the protein and transcript level in response to dietary yeast cell wall extract. The calreticulin-like protein was identified as a possible biomarker for yeast-derived functional feeds since it showed the most consistent change in expression in both the mucus proteome and skin transcriptome. The discovery of such a biomarker is expected to quicken the pace of research in the application of yeast cell wall extracts.

## Introduction

Functional feeds are used extensively in the aquaculture and agriculture sectors in order to protect livestock from pathogens and parasites, as well as a means to improve growth and feeding efficiency. These supplements offer an opportunity to the industry to reduce use of antibiotics and chemotherapeutants, which generate environmental sustainability concerns. A greater understanding of how these functional feeds might impart their benefits would allow for more evidence-based diet formulation. The aim of this study was to assess if one such functional feed, yeast cell wall extract (YCW), caused a change in components of the proteome of the salmon skin mucus that could be used as biomarkers for the response of salmon to dietary YCW.

Functional feeds contain both digestible and non-digestible components, and include probiotic and prebiotic supplements, nucleotides, vitamins, immunostimulants and algal/plant extracts [1]. Prebiotic supplements are non-digestible feed ingredients, usually purified from the yeast cell wall (YCW) or plant-derived, which profit the animal by promoting the growth of beneficial bacteria in the gut [2]. Mannanoligosaccharides (MOS), fructooligosaccharides (FOS), galactooligosaccharides (GOS) and hemicellulose are examples of the various available forms of prebiotics used in aquaculture. Numerous studies of prebiotic use in fish farms highlight the benefits such supplements lend to growth, feeding efficiency [3–6], immune response to bacteria [7–9], lysozyme activity [10,11], intestinal morphology [12–14] and gut microbiota [15–18].

Furthermore, YCW-derived supplements, such as β-glucans and MOS, have been linked with sea lice resistance [4,19]. However, research in this area has been hampered by high biological and technical variability across studies. Sea lice *Lepeophtheirus salmonis* (Krøyer, 1837) are ectoparasites of Atlantic salmon *Salmo salar* L., which disrupt the skin/mucus barrier by feeding on it. Although the infection rarely leads to death, it can compromise the defence mechanisms of the fish, leading to secondary infections. Sea lice control measures account for over £250m of expenditure per year to the global salmon farming industry [20]. Severe sea lice infestations on farms are of great environmental concern since chemotherapeutants can be detrimental to the surrounding ecosystem but, if left untreated, the sea lice may be transferred to the wild salmon populations [21,22]. In addition, there is growing concern that the sea lice are developing resistance to pesticides used for controlling the infection in farms [23].

YCW extracts have been shown to increase mucus production and/or goblet cell count in the intestine of various fish and other vertebrates [24–26]. A research area which has so far been overlooked is the effect of YCW extracts on the skin and mucus of fish, with only two reports in salmon [19,27]. Due to the nature of the sea lice infection, further study into this subject is required to elucidate the mechanism of the protection provided by dietary YCW extracts. In addition, discovery of a biomarker for the effect of dietary YCW in this tissue would be most useful to the aquaculture industry since it will hasten the pace of research, replacing the time-consuming and expensive sea lice challenges. Moreover, the skin and epidermal mucus of fish play an important role in the innate immune system by providing the first line of defence against pathogens and parasites. Apart from presenting a physical barrier to infection, the mucosal layer also provides powerful chemical protection. Mucus is mainly composed of large glycoproteins called mucins, which are responsible for the rheological and viscoelastic properties of mucus [28]. In addition, the mucus is also a complex mixture of elements of both the innate and acquired immune system, including antimicrobial peptides, complement, lectins, lysozyme and immunoglobulin, amongst others [29]. Changes in the skin mucus composition due to dietary YCW extracts may bring to light further benefits of such diets for farmed fish.

This paper reports the effect of a functional feed containing YCW extracts on the skin mucus proteome of Atlantic salmon, using two-dimensional sodium dodecyl sulphate polyacrylamide gel electrophoresis (2DGE). The analysis of skin mucus using 2DGE has been carried out in a variety of teleost species [30–32]. Results from this study show a number of proteins have altered expression in the mucus of the fish in response to inclusion of YCW extracts in the diet. A number of these proteins were identified following LC MS/MS and the transcript levels of the corresponding genes determined to assess whether the regulation of these genes was at the transcription level. These proteins are all worthy of further research to discover suitable biomarkers for YCW dietary modulation of skin mucus. An adequate biomarker assay will quicken the pace of research into functional feeds since it will complement or reduce the need for sea lice challenge experiments.

## Methods and Materials

### Animal husbandry and sample collection

Two feeding trials (Trial#1 and #2) were carried out at the Marine Environment Research Laboratory (Machrihanish, Scotland, United Kingdom). The experimental set up was composed of circular tanks (2 m diameter, 1,500 L volume) supplied with filtered sea water at ambient temperature (8–15°C) and salinity (28–34‰) via a flow-through system. Atlantic salmon (approx. 200 g) of a Scottish farmed strain were placed in each tank and fed on a standard commercial diet (BioMar Ltd, Grangemouth, United Kingdom). The diet was supplemented with 0.4% YCW and 0.1% FOS for half of the tanks, whereas the remaining tanks were fed YCW extracts-free basal diets and used as control. Fish were fed twice daily to apparent satiation and unconsumed food removed by an uplift system. Within each trial the control and YCW-containing diet were designed to be isonitrogenous and isolipidic. Full details of the diet compositions are given in S1 Table. Variations between the two trials are provided in Table 1.

**Table 1:** Details of feeding period and tank replicates for the feeding trials. The base diet was manufactured by Biomar and described in three terms: the product range name (CPK), the pellet diameter and the oil to protein ratio. The control and experimental diets were designed to be iso-nitrogenous and iso-lipidic. YCW = yeast cell wall extracts. FOS = fructooligosaccharides.

	Trial#1	Trial#2
**Dates**	September 2010	September 2011
**Base diet**	CPK 3mm 24/46	CPK 3mm 24/46
**Experimental diet**	0.4% YCW + 0.1% FOS	0.4% YCW + 0.1% FOS
**Feeding period**	7 weeks	4.5 weeks
**Tank replicates**	6	4
**Average fish weight, initial / g**	230	316
**Average fish weight, final / g**	350	350

Mucus/tissue sampling was carried out 7 weeks after the feeding trial was started for Trial#1 and 4.5 weeks for Trial#2 (Table 1). Three fish per tank were randomly selected and humanely killed by a sharp blow to the head followed by destruction of the brain. The study was approved by Institute of Aquaculture’s (University of Stirling) ethic committee and procedures carried out in accordance to the UK Animals (Scientific Procedures) Act 1986. Fish were monitored twice daily and no adverse effects were recorded throughout either of the trials. Immediately following death, mucus was collected by scraping one side of the fish using a small spatula from head to tail. The accumulated mucus (approx. 200 μL per fish) at the tail end of the fish was transferred into a 2 mL tube using a plastic Pasteur pipette and stored immediately on dry ice. For the gene expression studies, a skin sample (100mg) was taken from the opposite side of the fish from the dorsal area above the lateral line and stored in 1 mL RNAlater solution (Applied Biosystems, Warrington, UK). The skin samples were stored at 4°C for 24 h and then at -80°C until RNA extraction. For Trial#1, samples were also collected from the hindgut, cleaned of digesta and placed in RNAlater as for the skin samples.

Proteomic analysis was performed on samples collected during Trial#1. Skin samples from Trial#2 were used together with Trial#1 samples to further test the biomarker candidate molecules by qPCR.

### Proteomic samples preparation

For each mucus sample taken from individual fish from Trial#1 100 μL of protein extract was precipitated using the ReadyPrep 2-D Cleanup Kit (Bio-Rad Laboratories, Hertfordshire, UK) following the manufacturer’s guidelines. The final protein precipitate was solubilised in 100 μL re-swell buffer (7 M urea, 2 M thiourea, 4% 3-[(3-cholamidopropyl)dimethylammonio]-1-propanesulfonate (CHAPS), 0.3% dithiothreitol (DTT), 1% (v) IPG Buffer pH 3–10 (GE Healthcare). Complete solubilisation of the pellet was achieved by briefly vortexing the tubes followed by clarification by centrifugation at 14,500 x *g* for 15 minutes. The supernatant was then transferred to a clean 1.5 mL tube. The protein samples were initially analysed by 1-Dimensional sodium dodecyl sulphate polyacrylamide gel electrophoresis (SDS-PAGE) and Coomassie brilliant blue G-250 (CBB) (Fisher Scientific, Loughborough, UK) staining to determine the sample quality and to provide an estimate of the protein concentration.

### Two-dimensional gel electrophoresis

For this study, 12 preparative 2D gels were run, 6 gels per diet representing an individual fish from all the tanks used in the feeding trial. 20–25 μL of each sample was mixed with 200 μL of re-swell buffer and allowed to equilibrate at room temperature for 10 minutes. The treated protein extracts were centrifuged for 5 minutes at 14,500 x *g*. 200 μL of the supernatant was transferred to a re-swelling cassette and used to rehydrate 11 cm immobilised pH gradient (IPG) strip, with a linear pH4-7 gradient (Immobiline™ DryStrips, GE Healthcare, Amersham, UK). The rehydration process was carried out passively overnight. Isoelectric focusing was achieved using a Multiphor II electrophoresis unit (GE Healthcare) in three stages through a ramped voltage change (1 minute at 200 V, followed by 1.5 hours increasing from 200 V to 3,500 V and finally 7 hours at 3,500 V). The strips were reduced and alkylated using 10 mg mL^-1^ DTT and 25 mg mL^-1^ iodoacetamide, respectively. These solutions were made up in an equilibration buffer containing 0.05 M Tris, pH 6.8, 6 M urea, 30% glycerol and 10% SDS. The second dimension was performed on an AnykD Precast Criterion gel (Bio-Rad Laboratories) electrophoresed at 100 V for 35 minutes followed by 200 V for 35 minutes. Staining was attained by first fixing the gels overnight (50% ethanol, 2% phosphoric acid), then rinsing 3-times with MilliQ water and staining with CBB in an equilibration solution (34% methanol, 17% ammonium sulphate, 2% orthophosphoric acid). The gels were scanned as 16-bit grey images using an Image Scanner III (GE Healthcare).

Quantitative analysis of the 2D protein profiles was performed using Progenesis SameSpots v4.5 (Nonlinear Dynamics Limited, Newcastle upon Tyne, UK). The 2D protein profile showing optimal image quality and spot resolution was selected as a reference image and all of the other 2D protein profiles were aligned to this automatically; the automatic alignment of the gel images was manually confirmed and edited as required. Subsequent spot detection, volume normalization, background correction and differential expression analysis was performed using in-built routines from Progenesis SameSpots.

### LC-MS/MS analysis

Proteins chosen for identification by mass spectrometry were selected according to the magnitude of change in intensity across treatments and the statistical significance. The selected spots were manually excised from a dry-gel and in-gel trypsin digestion was carried out by Investigator ProGest robotic workstation (Genomic Solutions Ltd., Huntington, UK), following the method described by Shevchenko *et al*. [33] adapted for CBB-stained gels. Briefly, the excised protein was reduced and S-alkylated by incubating with DTT at 60°C for 20 minutes and with iodoacetamide at 25°C for 10 minutes. Trypsin digestion was achieved by incubating with sequencing grade trypsin (Promega, Southampton, UK) at 37°C for 8 hours. The resultant peptide extract was then dried by rotary evaporation (SC110 Speedvac; Savant Instruments, Holbrook, NY, USA) and dissolved in 0.1% formic acid for liquid chromatography tandem mass spectrometry (LC-MS/MS) analysis on electrospray ionisation (EIS)-ion trap instrument.

Liquid chromatography was performed on an UltiMate 3000 LC System (Dionex Ltd., Camberley, Surrey, UK) using a Monolithic Capillary Column (200 μm i.d. x 5 cm; Dionex). Eluent A was constituted from 3% acetonitrile in water containing 0.05% formic acid, whereas eluent B was made up from 80% acetonitrile in water containing 0.04% formic acid with a gradient of 3–45% B over 12 minutes at a flow rate of 2.0 μL min^-1^. Peptide solutions were analysed in an HCTultra PTM Discovery System (Bruker Daltonics Ltd., Coventry, UK). Peptide fragment mass spectra were obtained in data-dependent AutoMS(2) mode with a scan range of 300–1,500 m/z, three averages and up to three precursor ions selected from the MS scan 100–2,200 m/z. Precursors were actively excluded within a 1.0-min window, and all singly charged ions were excluded. Peptide peaks were detected and de-convoluted automatically using the incorporated data analysis software.

### Protein identification

Mass lists in the form of Mascot Generic Files were used as input for Mascot MS/MS ion searches of the NCBI database using the Matrix Science web server, www.matrixscience.com [34]. In addition, Mascot searches were also carried out for all samples on an in-house database consisting of expressed sequence tags (ESTs) and generated contigs of salmonids downloaded from the Genomic research on all salmon consortiums [35]. The search parameters used were: Enzyme = Trypsin, Max. Missed cleavages = 1; Fixed modifications = Carbamidomethyl (C); Variable modifications = Oxidation (M); Peptide tolerance ± 1.5 Da; MS/MS tolerance ±0.5 Da; Peptide charge = 2+ and 3+; Instrument = ESI-TRAP. If the top match in the salmonid EST/contig database was to a non-annotated sequence, it was run through a BLASTx search against NCBI non-redundant protein sequences to identify the protein.

### RNA purification and cDNA synthesis

Total RNA was extracted from skin samples of 10 fish per diet from both Trial#1 and #2 using TRI reagent (Sigma), as instructed by the manufacturer. RNA was also extracted in the same manner for the Trial#1 gut samples (n = 6). The concentration and quality of the RNA was assessed by both the ND-1000 Nanodrop Spectrophotometer (Labtech Inc., East Sussex, UK) and the Agilent 2100 Bioanalyzer (Agilent Technologies, Cheshire, UK). RNA (2 μg) was used for cDNA synthesis using Bioscript reverse transcriptase kit (Bioline, London, UK) and 0.2 μg of Random Hexamer Primer (Thermo Scientific, Northumberland, UK). The mixture was incubated at 70°C for 5 minutes and then left to rest on ice for 2 minutes. The master mix for 25 μL reactions was prepared as described in the kit protocol. The tubes were incubated at 25°C for 10 minutes, 45°C for 60 minutes and 72°C for 10 minutes. The cDNA was diluted five-fold using 1x Tris-EDTA (TE) buffer (Sigma).

### Real-time qPCR

Primer pairs (Table 2) were designed for the genes corresponding to candidate proteins that were found to be differentially expressed in the 2D gel analysis following the feeding trial. For the primer design, at least one of the primers was located on an intron/exon junction to avoid amplification of genomic contaminations. The position of the splice sites was determined by the publically available draft salmon genome.

**Table 2:** Primer sequences, together with their optimum annealing temperature and product size, used for the gene expression studies in Atlantic salmon skin cDNA.

Gene		Primers	Sequence (5’-3’)	PS*/ bp	Ann.^†^ T/°C	E% ^§^	R^2^^¤^
Name	Accession						
**Elongation factor 1α**	**BT060384**	EEF1a_F	CAAGGATATCCGTCGTGGCA	313	61	102	0.990
		EEF1a_R	ACAGCGAAACGACCAAGAGG				
**β-actin**	**BT047241**	ACTB_F	ATGGAAGATGAAATCGCCCC	239	61	102	0.986
		ACTB_R	TGCCAGATCTTCTCCATGTCG				
**Calreticulin-like**	**BT072764**	CALRL_F	CCGCTGACTCTACCATCTACAA	192	62	101	0.993
		CALRL_R	CTTGTCATCTTCCTCCTGTTTC				
**Calreticulin**	**BT044674**	CALR_F	AACATTGGAGTGTTGGGTCTGG	173	67	102	0.996
		CALR_R	TTTCCTCTCCTCCTCCTCCTGT				
**60S acidic ribosomal**	**BT059903**	RPLP0_F	GTTGCTGCCTCACATCAAAG	211	59	87	0.990
**protein P0**		RPLP0_R	AGATCTTGGTGGTGATGCC				
**Hemopexin-like protein**	**Z68112**	HPXL_F	AGAGGCCACCACTTCCTGGA	225	66		
		HPXL_R	TCCACTCCCAGCACCTCCTT				
**40kDa peptidyl-prolyl**	**BT046523**	PPID_F	GAAGCCATGTGTAATTGCTGAG	240	62	102	0.990
**cis-trans isomerase**		PPID_R	CAGATACCTGAGAGCTTTGGAGT				
**Keratin, type I**	**AJ427868**	KRT13_F	GAAGGAGGAGCTCATCTATCTCAAG	209	60	99	0.997
**cytoskeletal 13**		KRT13_R	GTCTCCGTCTTGGTCTGGAA				
**Glyceraldehyde-3-**	**BT045621**	GAPDH_F	ATGACCACAGTCCACGCCTA	169	65	81	0.991
**phosphate dehydrogenase**		GAPDH_R	ATGCCAGTCAGCTTGCCGTT				

* PS = product size

^†^Ann. = annealing

^§^ E% = primer efficiency

^¤^ R^2^ = coefficient of determination.

The qPCR assay was performed with Immolase DNA Polymerase (Bioline). The master mix was prepared as described previously [36]. The qPCR reaction was set up by mixing 16 μL of this master mix with 4 μL diluted cDNA in a well of a 96-well plate. Each reaction was run in triplicate. A serial dilution was used to determine primer amplification efficiency and to generate a standard curve for calculations of expression. This was prepared by mixing an equimolar amount of six different cDNA samples (three from each dietary treatment) and serially diluting it with TE buffer 10-fold 5 times. Negative (no template) controls were also included in each plate. The temperature programme was set up as follows: 95°C for 10 min, followed by 35–40 cycles of 95°C for 30 s, annealing at 59–67°C depending on primer (Table 2) for 30 s, 72°C for 30 s and finally, 75°C for 5 s after which fluorescence was measured. A melting curve was performed between 70°C to 95°C, to confirm amplification of a single product. The reaction was normalised using two house-keeping genes, elongation-1 alpha and β-actin.

### Data and statistical analysis

Statistical evaluation of the normalised spot intensities from the 2D gels was carried out on Progenesis SameSpots. The analysis was done by comparison of the gels according to dietary treatment and hence, values for fold change and Analysis of Variance (ANOVA) p-values were generated for each spot. At this point, protein spot matches were evaluated again and, where necessary, spots were merged or split.

Using the data for the serial cDNA dilution and the Ct values, arbitrary units for the expression of genes of interest and house-keeping genes in every skin sample were calculated. The arbitrary units of the genes of interest were normalized using that of the housekeeping genes. The Shapiro-Wilk test was used to check whether the arbitrary units fit a normal distribution curve. When the data were non-normal, appropriate transformations were applied; most often log10 transformation. Student’s t-test was used to assess whether the mean expression in the skin of control and prebiotic-fed fish is significantly different. Data analysis was carried out on SPSS Statistics 20 (IBM, Hampshire, UK). Statistical significant differences were indicated at *P*<0.05 unless stated otherwise.

### Calreticulin phylogenetic tree

In order to identify the salmon calreticulin isoforms, the NCBI and ENSEMBL databases were queried to discover other isoforms of salmon and also well-characterised calreticulin proteins of other vertebrates. The calreticulin sequences were aligned and a phylogenetic tree was constructed on MEGA5 using the neighbour-joining method [37].

## Results

### Proteomic analysis

**Fig 1:**
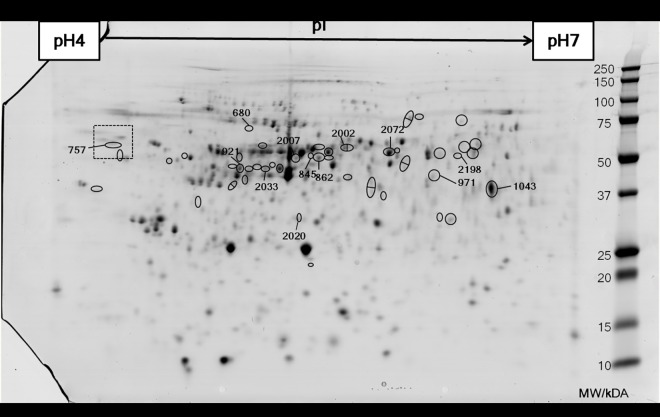
Representative gel of a two-dimensional SDS-PAGE of the epidermal mucus of Atlantic salmon. The first dimension was run on a pH4-7 IPG strip and the second dimension was run on an Anykd Criterion gel. Molecular weights in kDa are denoted on the right side of the image as indicated by Precision Plus Dual Color protein standards (Bio-Rad). The circled spots represent those that show significant differential expression between dietary treatments as given by SameSpots analysis (n = 6, p<0.05). The spots that are circled and numbered refer to those that were subsequently identified by LC-MS/MS. The boxed area refers to Fig 2 where this part of the image has been magnified.

The 2D gels were of good quality and demonstrated consistent protein spot resolution and all 12 were included in the analysis. The 2D protein profiles were matched, as described in Methods, and after filtering out poorly resolved areas of the protein profile and also any protein spots that were too faint and/or small, 705 spots were carried forward for analysis. Of these, 49 protein spots demonstrated changes in abundance due to the dietary treatments (Fig 1). The p-value of these spots were in most cases < 0.05 in the ANOVA. However, spots with a p-value < 0.10 were also included, particularly those that had a high-fold change and were protein spots that had been observed to change in abundance in another similar feeding trial in which the experimental diet included a different YCW product but lacked the 0.1% FOS (S1 File).

Thirteen protein spots out of the 49 significant ones were chosen for further identification by LC-MS/MS (Table 3). These 13 spots were chosen on the basis of the magnitude of their change and level of statistical significance. Eight of the identified proteins were salmon keratins, which are non-secreted structural molecules expressed in the skin of fish typically found in keratinocytes, mesenchymal cells or cytoplasm (in the case of type II keratins) [38,39]. Among the identified proteins, only calreticulin and hemopexin-like protein have signal peptides and are expected to be secreted via the classical secretion pathway.

**Table 3:** Differentially expressed proteins in the skin of Atlantic salmon fed prebiotic dietary supplements, as identified by two-dimensional electrophoresis. The relevant spots were cut out from the gel and sequenced by LC-MS/MS, after which the peptides were identified by a MASCOT search. The spot numbers refer to Fig 1, MW stands for molecular weight and fold change represents the expression level in the experimental fish group as compared to the control.

Spot #	Accession number	Protein ID	MW/Da	pI	MASCOT results				Fold change	p-value
					Matched Peptides		Sequence coverage/%	Score		
680	GI:1848139	Hemopexin-like protein (*Oncorhynchus mykiss*)	76,688	6.25	3	5		173	+1.8	0.048
757	GI:224613524	Calreticulin precursor (*Salmo salar*)	30,030	4.51	15	34		691	+1.3	0.080
2020	GI:209736184	60S acidic ribosomal protein P0 (*Salmo salar*)	31,083	9.10	35	62		1002	+1.3	0.017
2198	GI:185133596	Type I keratin E7 (*Oncorhynchus mykiss*)	23,095	5.11	11	19		240	-2.3	0.023
845	GI:185135325	Keratin, type I cytoskeletal 13 (*Oncorhynchus mykiss*)	71,984	7.72	8	21		277	-2.1	<0.001
921	GI:185135325	Keratin, type I cytoskeletal 13 (*Oncorhynchus mykiss*)	71,984	7.72	21	30		824	-2.0	0.003
2002	GI:185132221	Type II keratin E1 (*Oncorhynchus mykiss*)	97,540	8.68	6	9		350	-2.0	0.015
2033	GI:185135325	Keratin, type I cytoskeletal 13 (*Oncorhynchus mykiss*)	71,984	7.72	39	44		1215	-1.8	0.021
2007	GI:185135325	Keratin, type I cytoskeletal 13 (*Oncorhynchus mykiss*)	71,984	7.72	22	23		798	-1.7	0.005
2072	GI:185132221	Type II keratin E1 (*Oncorhynchus mykiss*)	97,540	8.68	36	34		1224	-1.7	<0.001
862	GI:185133596	Type I keratin E7 (*Oncorhynchus mykiss*)	31,541	5.04	12	16		330	-1.5	0.043
971	GI:209730456	40 kDa peptidyl-prolyl cis-trans isomerase (*Salmo salar*)	31,499	5.42	18	50		682	-1.5	0.016
1043	GI:185132746	Glyceraldehyde-3-phosphate dehydrogenase (*Oncorhynchus mykiss*)	51,406	8.90	47	56		853	-1.4	0.027

**Fig 2:**
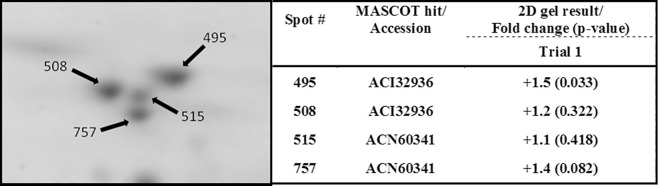
Magnification of the gel image in the area where the calreticulin spot (no.757) was located in Fig 1. The table indicates the identities the other protein spots migrating in this region of the profile along with their fold-change and p-value from the SameSpots analysis of mucus samples from Trial#1.

**Fig 3:**
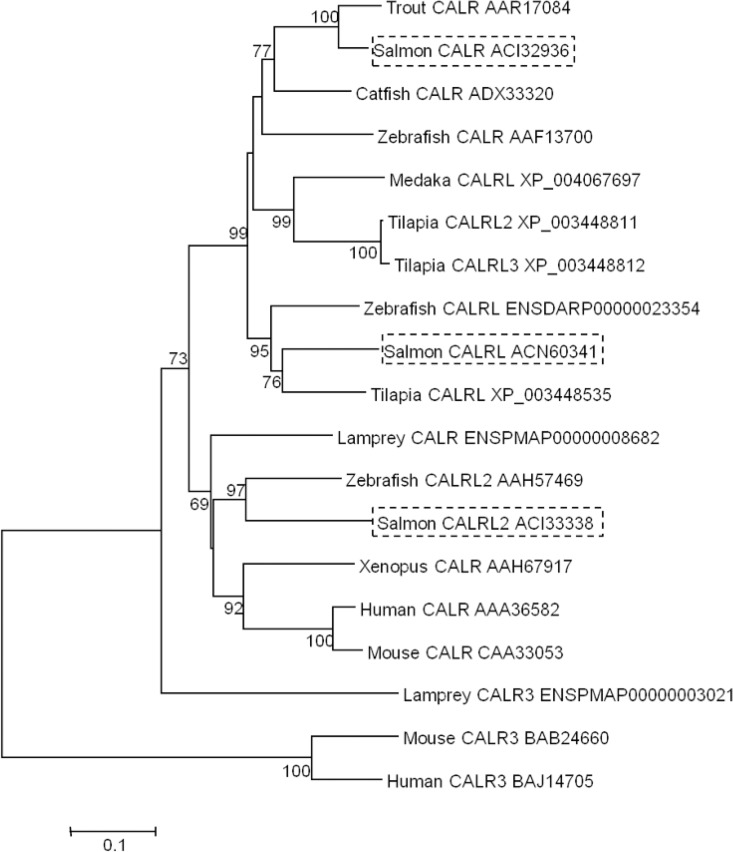
Phylogenetic trees showing the evolutionary relationship of the calreticulin family of proteins in vertebrates, rooted by the mammalian calreticulin-3. The tree was used to name the calreticulin isoforms of *Salmo salar*. The tree was constructed using multiple alignments of the vertebrate calreticulin. The neighbour-joining method in MEGA 5 [37] was selected. The percentage of replicate trees in which the associated taxa clustered together in the bootstrap test (10,000 replicates) is shown next to the branches [40]. The salmon sequences are boxed. Each entry is described by the species common name and protein name, followed by the Genbank or Ensembl accession number.

Calreticulin was studied further since this protein is involved in pathways that could potentially be affected in mucosal layers following feeding with YCW extracts, such as protein glycosylation and stimulation of the innate immune system. Four spots in close proximity were identified by LC-MS/MS as two isoforms of salmon calreticulin (ACI32936 and ACN60341) (Fig 2). A third isoform of calreticulin was identified (ACI33338) from the NCBI protein database. The phylogenetic tree aided the classification of the identified salmon calreticulins as calreticulin CALR (ACI32936), calreticulin-like CALRL (ACN60341) and calreticulin-like 2 CALRL2 (ACI33338), by comparison with the homologous zebrafish calreticulin isoforms (Fig 3).

### Gene expression analysis

**Fig 4:**
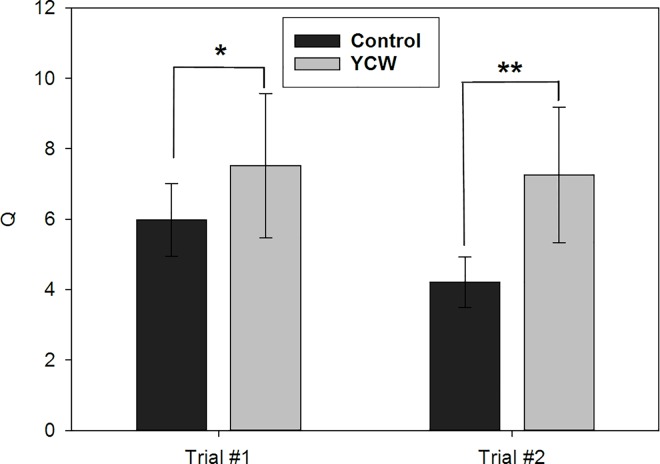
Effect of dietary YCW extracts on *calrl* expression in the skin of Atlantic salmon during two independent feeding trials. Fish were fed either a control diet (Control) or the same base diet supplemented with 0.4% YCW for 4–7 weeks. Expression is given as a ratio of the arbitrary unit for *calrl* to the arbitrary units of two reference genes, elongation factor 1-alpha and beta-actin. Data are present as Means ± SEM (n = 9). Statistical significance was calculated by independent sample t-test, after checking for normality and outliers. One star (*) denotes p<0.10 and two stars (**) denotes p<0.05.

**Fig 5:**
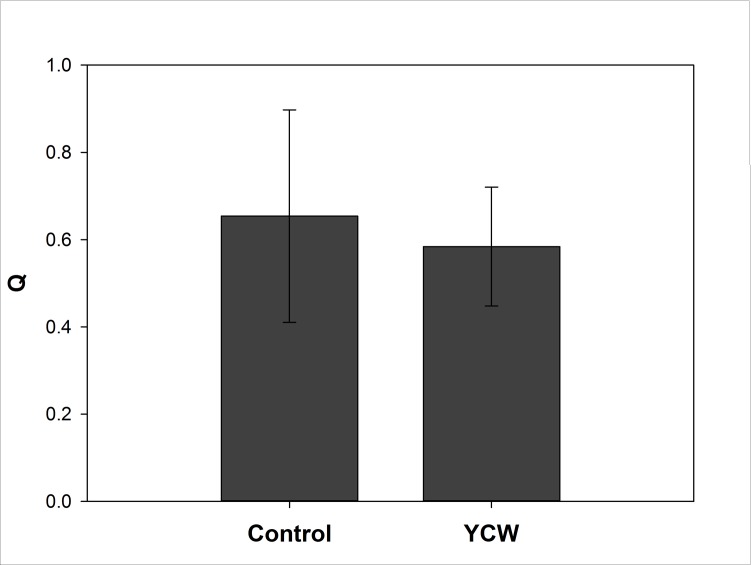
Effect of dietary YCW extracts on *calrl* expression in the gut of Atlantic salmon during Trial#1. Fish were fed either a control diet (Control) or the same base diet supplemented with 0.4% YCW. Expression is given as a ratio of the arbitrary unit for CALRL to the arbitrary units of two reference genes, elongation factor 1-alpha and beta-actin. Data are present as Means ± SEM (n = 6). The expression of CALRL is not significantly different between diets (p>0.05), as established by independent sample t-test after checking for normality and outliers.

For proteins that had shown changes in abundance in skin mucus in Trial#1, the corresponding gene transcript levels were determined in this trial and a second, independent trial (Trial#2). The majority of the genes assayed using qPCR were not significantly altered in expression across the dietary treatments in either of the trials (Table 4). The expression of hemopexin-like gene was not detected in the skin in any sample. In contrast, *calrl*, but not *calr*, was up-regulated in both Trial#1 and #2 in fish fed the YCW extract diet (Fig 4), thus, showing potential as a suitable biomarker for dietary YCW extracts. However, no significant changes in the expression of either of the calreticulin isoforms mRNAs were detected in the gut in fish from Trial#1 (Fig 5).

**Table 4:** Validation of proteomic results by analysing the gene expression of the skin mRNA corresponding to the respective proteins. Fold changes at the proteomic level was calculated on SameSpots using normalised spot volume data of two-dimensional gels (2DGE). Gene expression fold changes were calculated from C_t_ values of qPCR assays by deriving arbitrary units using the standards graph equation and normalising using two housekeeping genes, EF-1α and β-actin. (n = 9).

	Trial#1		Trial#1	Trial#2
Primer	2DGE fold change	*P* value	qPCR fold change	
**CALR**	+1.2	0.40	n.s	n.s.
**CALRL**	+1.3	0.08	+1.26^†^	+1.72*
**GAPDH**	-1.4	0.03	n.s.	n.s.
**HPXL**	+1.8	0.05	n.e.	n.e.
**KRT13**	-1.8	0.02	n.s.	n.s.
**PPID**	-1.5	0.02	n.s.	n.s.
**RPLP0**	+1.3	0.02	n.s.	+1.39*

^†^ p<0.075

* p<0.05

n.s. = not significant; n.e. = not expressed.

## Discussion

In the present experiment, the effect of dietary YCW extracts on the skin mucus proteome of Atlantic salmon was studied using 2D gel electrophoresis. Results show that multiple proteins in the skin mucus of Atlantic salmon exhibited different expression levels between diets, with the majority of these being down-regulated in the YCW-fed individuals. These proteins were all considered to be potential biomarkers. However, gene expression analysis of these molecules on the skin cDNA from two feeding trials was required to verify CALRL as the most suitable biomarker candidate since its up-regulation was manifested in both trials. The levels of significant probability were not particularly strong (P = 0.05–0.08) for a change in CALRL/*carl* in Trial#1 and #2. However, they were comparable with a similar pilot feeding trial in which CARL was increased 1.5-fold (P < 0.03) in salmon fed a diet containing a different YCW product and lacking FOS (S1 File). Though the differences in the diet compositions prevent direct comparisons, CALRL/*carl* was increased in abundance in mucus or skin in salmon fed diets containing YCW across all three independent trials.

Down-regulation of genes due to dietary YCW extracts has been previously reported in microarray studies of Atlantic salmon liver [1] and rainbow trout gut and gills [41]. YCW extracts improve growth and feeding efficiency ratios in flounder [11] and snakehead [8]. This suggests that the fish fed YCW extracts are investing less energy on protein turnover in order to utilise it for growth, but such a postulation was not investigated in our study. Glyceraldehyde-3-phosphate dehydrogenase (GAPDH), occasionally used as a reference gene, was amongst the down-regulated proteins in this experiment. This result supports previous studies that GAPDH is not suitable for use as a housekeeping molecule since it participates in a variety of cellular processes [42]. Peptidyl-prolyl cis-trans isomerase is notable because peptidyl-prolyl cis-trans isomerase A was down-regulated in a LC-MS/MS proteomic study on the mucus of salmon fed unspecified prebiotic supplements [26]. The MASCOT search of the peptide sequences for spot 971 indicate that the protein in this experiment was more similar to peptidyl-prolyl cis-trans isomerase D (PPID), also known as cyclophilin D which is involved in mitochondrial permeability [43] as well as suppression of apoptosis [44] in mammalian models. Further investigation is required to deduce its function in fish and potential participation in the immunomodulatory effect of YCW extracts. However, it should be noted that though PPID was up-regulated at the protein level in Trial#1, up-regulation at the transcript level was not observed in either Trial#1 or Trial#2.

Three different keratins were also down-regulated. Keratins are a large family of proteins which make up most of the intermediate filament [45], responsible to maintain the cell structure. Most keratins are associated with epithelial cells although others are found in mesenchymal tissue. Since the epidermal layer of fish skin is situated on top of the scales [46], parts of it would have been present in the mucus samples as the collection method was not sufficiently selective. This explains the presence of non-secreted intracellular proteins in the mucus. Type II keratins are also associated with scales [40], which were also present in the mucus samples. The down-regulation of such proteins could be due to inconsistencies in the presence of scale/epidermal cells in the samples. Whether these inconsistencies in sampling are actually related to dietary-induced physicochemical changes in the fish mucus is unclear, but no difference in keratin transcript levels was observed in any of the trials.

From the identified mucus proteins, only calreticulin and hemopexin-like protein have a signal peptide (i.e. are secreted molecules). This implies that the rest of the identified proteins are either intracellular proteins found in the skin epidermis, or are secreted into the mucus via alternative secretion pathways. Hemopexin-like protein exhibited the highest positive fold-change in the study. Hemopexin is expressed in the liver and then secreted into blood serum [47] and, thus, no gene expression was detected in any of the skin samples. Hemopexin provides protection to the cells from oxidative damage and toxicity caused when haemoglobin is released from damaged erythrocytes [48]. Hemopexin is part of the acute-phase response during bacterial infection by binding iron, hence preventing its use by the bacteria [30,49]. Further research into the function of this protein in fish is required in order to deduce its role in mucus during the response to YCW extracts.

Calreticulin showed greatest potential as a biomarker for YCW-derived functional feed, as it was up-regulated by a small but significant fold change in the proteomic study and this up-regulation was also replicated when studying the gene expression in the skin from fish of the same trial. Importantly, it was also up-regulated in the skin cDNA samples of an independent feeding trial, Trial#2. This proteomic study has brought to light two different isoforms of salmon calreticulin in the mucus and additional database searches have put forward a third isoform which was not identified on the gels. Specifically, only the calreticulin-like (CALRL) isoform was up-regulated due to dietary YCW extracts. Expression of this candidate biomarker molecule was also explored in the gut cDNA samples as another mucosal tissue with a more direct association with dietary intake. However, results show that the effect appears be skin-specific since no changes were detected in the gut.

Calreticulin is a multi-functional protein and thus it is challenging to deduce the physiological implications of its differential expression in Atlantic salmon. Calreticulin is a soluble protein found in the lumen of the endoplasmic reticulum [50]. It is also found in the extracellular matrix and it has been linked to mineralisation in teeth and bone formation due to its high affinity to calcium ions [51]. As a chaperone protein, calreticulin binds to glycoproteins in the endoplasmic reticulum and is involved in their folding and degradation [52]. In the context of this study, it is particularly interesting that calreticulin is directly involved in the synthesis of mucins [53], the main macromolecule of mucus. Yeast-derived prebiotic dietary supplements have been linked to an increase in mucus layer thickness [54] and goblet cell number [5,25] in the gut. Furthermore, mucin 5B was up-regulated by three-fold in the skin mRNA of common carp *Cyprinus carpio* fed a β-glucan, a type of YCW extract [55]. It was not possible to examine the mucin content in the mucus during this experiment since the size of gel-forming mucins [56] is beyond the range of sizes resolved on 2D gels.

Calreticulin has been found modulated in several previous proteomic studies in fish. It was decreased in rainbow trout liver following exposure to verpamil, a calcium ionophore which is toxic to fish [57]. Secondly, in medaka *Oryzias melastigma*, calreticulin protein levels were decreased in gill after treatment with brevetoxin-1, a fish toxin associated with toxic algal blooms [58]. In a recent study, calreticulin was over expressed in the proteome of the distal intestine of Atlantic salmon 24 h after inducing inflammation [59]. Up-regulation of three isoforms of calreticulin was recorded in the channel catfish *Ictalurus punctatus* after iron-dextran treatment and/or bacterial challenge [60]. This response has highlighted the possible participation of calreticulin in the innate immune response of fish via binding to components of the lectin [61] or complement [62] pathways. On binding, calreticulin induces a change in the conformation of these molecules, leading to elimination of apoptotic cells and other immune complexes. Both lectin and complement must be immobilised in order to bind to calreticulin and, in the case of mannan-binding lectin, this is achieved by binding to mannan. The YCW extract used in this study contained >20% w/v mannanoligosaccharide (MOS) with the remainder mainly comprised of proteinaceous material, but also other cell wall fractions including β-glucans and cellular constituents such as nucleotides. Alternatively the main dietary constituents of MOS and FOS, which are polymers of the mannan and fructose sugars respectively, can be substrates for bacterial growth within the gut, thereby selectively modifying the microbial composition, which in turn may have elicited the skin mucus response.

In the gene expression studies using skin cDNA as template, only CALRL exhibited a consistent dietary effect in both its proteomic expression in the epithelial mucus and transcriptomic expression in the skin. The rest of the studied proteins/genes did not follow such a trend. This could because biological variation was quite high at both the proteomic and transcriptomic level, although in the latter it was even more so. Attempts were done to counteract this by using more biological replicates in the qPCR assays (9 replicates as opposed to 6 replicates in the proteomic work). In addition, one gene isoform may result in multiple protein spots on 2D gels due to post-translational modification that alter the pI of the protein, as was the case with the multiple spots for calreticulin and keratin isoforms. Transcriptomic effects are also more transient in comparison to protein turnover and thus, more changes will be detected at the protein level rather than at the gene expression level. Also, some proteins present in the mucus might not be secreted by the skin; a few proteins could form part of the mucus through leakage of the secondary circulation system [30]. This could have been the case of hemopexin-like protein, which was identified from the 2D gels but showed no expression in the skin mRNA. Hemopexin-like proteins are produced and expressed mainly in the liver; studies in teleost models support our findings that it is not expressed in the skin [63,64]. However, this protein must be transported to peripheral tissues, since 2D gels in the muscle [65] and gills [66] of rainbow trout have also detected it.

Limitations of 2D gels are posed by the image resolution, detection of low abundance proteins, those outside the pI range and very large or very small proteins. The use of skin mucus to detect dietary changes has merit as it is easy and quick to collect non-destructively from live animals and such assays would be highly beneficial to the aquaculture industry. However, it also makes this study more complex since its proximity to the surrounding environment brings about high biological variation across replicates. Nevertheless, our proteome-led approach did identify a few proteins whose expression was altered by dietary YCW and allowed us to further assess the validity of such biomarkers by targeted gene expression analysis across independently replicated feed trials.

In conclusion, this study has confirmed that dietary YCW extracts cause a change in the proteomic profile of Atlantic salmon skin mucus. CALRL was identified as a potential biomarker for research in YCW-derived diets and performance of salmon following different health challenges. While specific benefits of using YCW extracts in aquaculture are still being discovered, a biomarker would facilitate such studies by making them quicker and less costly. In addition, this study highlights the need of further research into the microbial changes in the gut of fish fed YCW extracts in order to discover the trigger behind proteomic and transcriptomic changes.

## Supporting Information

S1 File Pilot Feeding Trial. Details of a pilot feeding trial, including proteomic analysis.
(DOCX)

S1 Table Details of diets used in Trials #1 and #2. (DOCX)
