# Supplementary material for: Dietary Yeast Cell Wall Extract Alters the Proteome of the Skin Mucous Barrier in Atlantic Salmon (Salmo salar): Increased Abundance and Expression of a Calreticulin-Like Protein
Source: PLoS One. 2017 Jan 3;12(1):e0169075. doi: 10.1371/journal.pone.0169075 (PMC5207756; doi:10.1371/journal.pone.0169075)
Supplement: S1 File — Details of a pilot feeding trial, including proteomic analysis. (DOCX) [file pone.0169075.s001.docx]

**Supplementary Data S2:**

**Pilot feed trial to assess effect of Yeast Cell Wall Extract (YCW) on skin mucus proteome.**

**Methods**:

Salmon (initial weight 150 g) were fed either a basal control diet or a diet including 0.4% YCW. Both diets were designed to be isonitrogenous and isolipidic. After 4 weeks feeding on these diets, ten salmon per diet were humanely killed and mucus collected, as per the main paper. Mucus was prepared, gel electrophoresis performed and LC-MS/MS performed, essentially as reported in the main paper. Analysis was performed on 4 fish per diet, each fish ran on an individual gel.

**Results:**

The gels showed good quality and resolution (Figure S1) and thus were all included in the analysis (4 control-diet and 4 for YCW-diet). After filtering out artefacts, 807 spots were put forward for expression analysis from the Pilot Feed Trial gels. The analysis exposed 27 spots whose change in expression was significant (P<0.05), 22 of which were down-regulated proteins and 5 were up-regulated proteins. Eight of these proteins were identified by LC-MS/MS (Table S1). One of the up-regulated proteins was the calreticulin-precursor (GI: 209148412) that increased by 1.5-fold (P = 0.033) in response to the YCW-containing diet compared to the control diet.


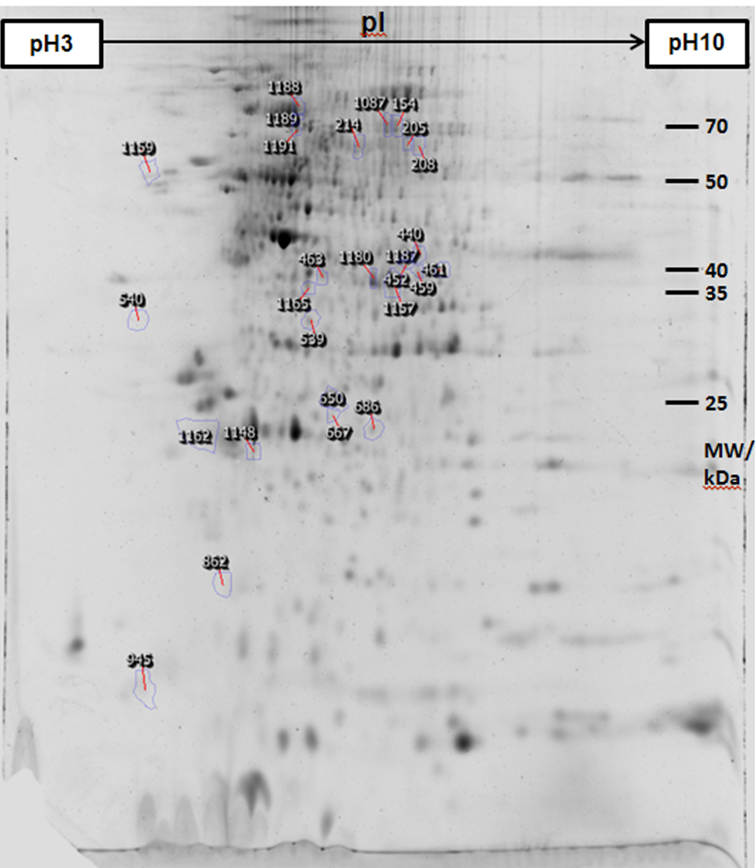


**Figure S1:** Representative gel of a Pilot Feed Trial two-dimensional SDS-PAGE which separates out the proteins found in the epidermal mucus of Atlantic salmon. The first dimension was run on a pH3-10 IPG strip, while the second dimension was run on a 12.5% polyacrylamide gel prepared in-house. Molecular weights (MW) in kDa are denoted on the right side of the image as inferred from the identified protein spots. The labelled spots represent those that show significant differential expression between dietary treatments as given by SameSpots analysis (p<0.05). The arrow points to the calreticulin-precursor spot.

Table S1 - Differentially expressed proteins in the skin mucus of Atlantic salmon fed 0.4% YCW supplements in the Pilot Feed Trial, as identified by two-dimensional electrophoresis. The relevant spots were cut out from the gel and sequenced by LC-MS/MS, after which the peptides were identified by a MASCOT search. The spot numbers refer to Figure S1, MW stands for molecular weight and fold change represents the expression level in the experimental fish group as compared to the control.

| Spot # | Accession number | Protein ID | MW/Da | pI | MASCOT results | | | Fold change | p-value |
| --- | --- | --- | --- | --- | --- | --- | --- | --- | --- |
|  |  |  |  |  | Queries matched | Sequence coverage/% | Score |  |  |
| 1159 | GI:209148412 | Calreticulin precursor (*Salmo salar*) | 47,726 | 4.33 | 4 | 36 | 681 | +1.5 | 0.033 |
| 1191 | GI:348538728 | Plastin-3 (*Oreochromis niloticus*) | 70,070 | 5.28 | 4 | 17 | 497 | -1.7 | <0.001 |
| 650 | GI:220679252 | Glutathione S-transferase M (*Danio rerio*) | 26,444 | 6.03 | 6 | 31 | 376 | -1.7 | 0.004 |
| 1087 | GI:213511480 | Transketolase-like protein 2 (*Salmo salar*) | 68,897 | 6.18 | 6 | 35 | 763 | -1.7 | 0.016 |
| 463 | GI:223647378 | F-actin-capping protein subunit alpha-1 (*Salmo salar*) | 32,989 | 5.41 | 5 | 37 | 293 | -1.6 | 0.036 |
| 1165 | GI:225715630 | Pyruvate dehydrogenase E1 subunit beta, mitochondrial precursor (*Salmo salar*) | 39,797 | 5.52 | 4 | 29 | 523 | -1.4 | <0.001 |
| 154 | GI:213511480 | Transketolase-like protein 2 (*Salmo salar*) | 68,897 | 6.18 | 7 | 47 | 1004 | -1.4 | 0.006 |
| 1157 | GI:209155302 | Glyceraldehyde-3-phosphate dehydrogenase (*Salmo salar*) | 36,464 | 6.06 | 6 | 51 | 717 | -1.3 | 0.032 |
