# Supplementary material for: Dietary Yeast Cell Wall Extract Alters the Proteome of the Skin Mucous Barrier in Atlantic Salmon (Salmo salar): Increased Abundance and Expression of a Calreticulin-Like Protein
Source: PLoS One. 2017 Jan 3;12(1):e0169075. doi: 10.1371/journal.pone.0169075 (PMC5207756; doi:10.1371/journal.pone.0169075)
Supplement: S1 Table — (DOCX) [file pone.0169075.s002.docx]

**Supplementary Table S1:**

**Diet compositions for Trial #1 Diet compositions for Trial#2**

| Component (by weight) | Control | Experimental |
| --- | --- | --- |
| Fish meal | 46 | 46 |
| Vegetable proteins | 26 | 26 |
| Wheat | 7 | 7 |
| Fish oil | 21 | 21 |
| Vitamineral mix | 0.53 | 0.53 |
| Yeast cell wall (YCW) | 0 | 0.4 |
| Fructooligosaccharides (FOS) | 0 | 0.1 |
| **Calculated Proximate Content (%, w/w)** |  |  |
| Oil | 26 | 26 |
| Protein | 43 | 43 |

| Component (by weight) | Control | Experimental |
| --- | --- | --- |
| Fish meal | 52 | 52 |
| Vegetable proteins | 20 | 20 |
| Wheat | 9.4 | 9.4 |
| Fish oil | 19 | 19 |
| Vitamineral mix | 0.5 | 0.5 |
| Yeast cell wall (YCW) | 0 | 0.4 |
| Fructooligosaccharides (FOS) | 0 | 0.1 |
| **Calculated Proximate Content (%, w/w)** |  |  |
| Oil | 24 | 24 |
| Protein | 46 | 46 |
